# The effect of shame on endothelial function: discussing potential mechanisms and future directions

**DOI:** 10.1113/EP091191

**Published:** 2023-04-05

**Authors:** Ellen C. McGarity‐Shipley, Lindsay A. Lew, Jacob T. Bonafiglia, Kyra E. Pyke

**Affiliations:** ^1^ Cardiovascular Stress Response Laboratory, School of Kinesiology and Health Studies Queen's University Kingston Ontario Canada; ^2^ Muscle Physiology Lab, School of Kinesiology and Health Studies Queen's University Kingston Ontario Canada

In their letter to the editor, Craig and colleagues discuss a paper that we recently published (McGarity‐Shipley et al., 2022) in which we found that shame, a specific form of psychosocial stress, negatively impacted endothelial function but surprisingly did not elevate cortisol or the pro‐inflammatory marker soluble tumour necrosis factor‐α receptor 2 (sTNFαRII). To explain this phenomenon, Craig and colleagues hypothesize various alternative mechanisms that may underlie shame‐related reductions in endothelial function such as increased levels of noradrenaline, adrenaline, angiotensin‐II and β‐endorphins. We thank Craig and colleagues for their interest in our research and, in response, we would like to comment on some of their postulates.

While we agree with the potential mechanistic explanations put forth by Craig and colleagues, we want to highlight an important distinction for the discussion of psychophysiological mechanisms as they pertain to the impact of psychological stress on human physiology. ‘Stress’ is typically defined in a very general way as a systemic reaction to a challenge. However, as discussed in our original article (McGarity‐Shipley et al., 2022), previous researchers have put forth the integrated specificity model suggesting that different kinds of stress influence human physiology differently. Shame, a socially specific form of stress that comes from a decreased sense of social value (e.g., after being humiliated during a public speaking event), may have different impacts on human physiology than the stress that comes from responding to a physical threat (e.g., running out of a burning building). Therefore, it is likely that for us to understand the full picture of how stress alters human physiology and health, the effects of different kinds of stress and stress‐inducing protocols need to be appreciated and explored.

We agree with Craig and colleagues that our shame protocol likely increased sympathetic nervous system activity since there was an increase in mean arterial pressure after the shame protocol compared with the control protocol. Coinciding with this rise in sympathetic activity, we also agree that adrenaline, noradrenaline and angiotensin‐II may have increased and played a role in the reduction in endothelial function following our shame protocol. These mechanisms should therefore be explored in future investigations. Regarding Craig and colleagues’ suggestion to incorporate sympathetic blockade and blood pressure modulation into future studies, we would like to add that any potential experimental strategies outside of shame‐ or general stress‐induction protocols need to consider, and attempt to limit, a possible coinciding impact on participant emotions. For example, strategies that involve needles or participant discomfort should be used with caution to ensure that they are not confounding the effectiveness of the shame‐ or other stress‐induction protocol being used.

We welcome the discussion of opioid receptors and their potential role in the impact of shame on endothelial function. We agree that they likely play a role, although it may be complex involving actions of both μ_1_ and μ_3_ opioid receptors which may impair (Wilbert‐Lampen et al., 2006) or enhance (Stefano et al., 1995) endothelial function, respectively (Toda & Nakanishi‐Toda, 2011). If an enhancing effect of β‐endorphins on endothelial function tends to dominate, as suggested by Toda and Nakanishi‐Toda (2011) in their review, a decrease in β‐endorphins may explain the reductions in endothelial function following our shame protocol. This possibility is indicated by the significant decrease in positive affect after the shame protocol compared to the lack of change in the control protocol. To definitely test these hypotheses, we second the comments by Craig and colleagues that it would be helpful for future studies on shame and stress physiology to include measurement of β‐endorphins and nitric oxide bioavailability.

In conclusion, we share in Craig and colleagues’ curiosity surrounding the mechanisms of shame and stress physiology. We agree that further exploration of mechanisms including catecholamines and endorphins is important, as well as confirming findings, exploring the impacts of different forms of stress and stress protocols, and understanding the time course of psychophysiological effects. Relevant to our study (McGarity‐Shipley et al., 2022), investigating shame physiology specifically is important since shame is used in many different contexts (e.g., public health campaigns, healthcare practice, parenting and coaching). It is therefore very important that we understand how the use of shame, and its sources such as stigmatization, affect human physiology and health on small and large scales. We hope that our research will stimulate more shame physiology (and epidemiology) investigations, and that it contributes to promoting the importance of and understanding of psychophysiology overall. We are happy to be part of the conversation and we are eager for more research in this area.

## AUTHOR CONTRIBUTIONS

All authors have approved the final version of the manuscript and agree to be accountable for all aspects of the work. All persons designated as authors qualify for authorship, and all those who qualify for authorship are listed.

## CONFLICT OF INTEREST

No authors have any conflicts of interest to report.

## FUNDING INFORMATION

No funding was received to write this letter to the editor.
